# Genetically improved BarraCUDA

**DOI:** 10.1186/s13040-017-0149-1

**Published:** 2017-08-02

**Authors:** W. B. Langdon, Brian Yee Hong Lam

**Affiliations:** 1 Department of Computer Science, University College London, Gower Street, London, WC1E 6BT UK; 2 University of Cambridge Metabolic Research Laboratories, Addenbrooke’s Hospital, Cambridge, UK

**Keywords:** GPGPU, Parallel computing, Genetic improvement, Double-ended DNA sequence, Nextgen NGS

## Abstract

**Background:**

BarraCUDA is an open source C program which uses the BWA algorithm in parallel with nVidia CUDA to align short next generation DNA sequences against a reference genome. Recently its source code was optimised using “Genetic Improvement”.

**Results:**

The genetically improved (GI) code is up to three times faster on short paired end reads from The 1000 Genomes Project and 60% more accurate on a short BioPlanet.com GCAT alignment benchmark. GPGPU BarraCUDA running on a single K80 Tesla GPU can align short paired end nextGen sequences up to ten times faster than bwa on a 12 core server.

**Conclusions:**

The speed up was such that the GI version was adopted and has been regularly downloaded from SourceForge for more than 12 months.

## Background

### Why run bioinformatics on gaming machines

Bioinformaticians have seized the advantages of using computer graphics hardware (GPUs) [1], particularly those made by nVidia. Amongst other software tools, nVidia’s CUDA gives the ability to run C/C++ programs on nVidia GPUs. CUDA versions of several popular Bioinformatics applications have been written. In particular BarraCUDA [2], which aligns short noisy DNA sequences against one of the increasing number of reference genomes. It can align human DNA sequences when run on nVidia consumer GPU cards with more than 4 GB of memory, e.g. the GeForce GT 730. In fact it has been run on cards costing less than $100 up to $325 million super computers.

**Fig. 1:**
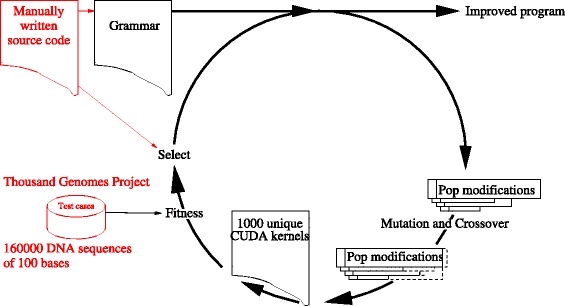
Major components of Genetic Improvement (GI)

Recently [3] we presented an approach in which a small part of the manually written code had been optimised by a variant of genetic programming [4, 5] to give a huge speed up on that part. (The raw graphics kernel can process well over a million DNA sequences a second ([3], Fig. 1)). The next section describes BarraCUDA and other programs (“Alternative tools” section) for aligning large numbers of next generation DNA sequences, whilst “Scalability” section considers factors affecting their performance and “Genetic improvement (GI)” section contains a very quick introduction to the genetic improvement of software (GI) technique used to create the current version of BarraCUDA. “Programs, DNA sequences and parallel operation under multi-core Unix” section gives details of the programs and DNA benchmarks. This is followed (“Results” section) by the overall performance changes genetic improvement [6–12] gives and comparison with bwa. (See particularly Table 3.)

## Introduction

### NextGen DNA sequence alignment

Both version of BarraCUDA and bwa use the Burrows-Wheeler algorithm. This requires the reference genome to be converted offline into an index file. The whole of the index must be kept in memory. Fortunately modern GPUs have high capacity and high bandwidth to their on-board memory (see last column in Table 1).

**Table 1:** Parallel computer graphics hardware

GPU	Compute level			MP	Total cores	Clock	Memory
GT 730	2014	£54	2.1	2 ×	48 = 96	1.40 GHz	4 GB 23 GB/s
Tesla K20	2012	£2905	3.5	13 ×	192 = 2496	0.71 GHz	5 GB 140 GB/s
Tesla K80^a^	2014	£6261	3.7	13 ×	192 = 2496	0.82 GHz	11 GB 138 GB/s

Fourth column is CUDA compute capability level. Each GPU chip contains 2 or 13 identical independent multiprocessors (MP, column 5). Each MP contains 48 or 192 stream processors (total column 7). Onboard memory size and bandwidth are given in the right most two columns. Technical report [36] has full details

^a^K80 is a dual GPU, Original total list price is followed by performance data for one half

Typically the Burrows-Wheeler algorithm scales linearly with the length of the DNA sequences to be looked up. This makes it more suitable for shorter sequences than for longer ones. Ideally sequences should not exceed 100 bp, however we have recently demonstrated BarraCUDA on paired end epigenetic data of 150 bp [13]. (Above 150 bp, BarraCUDA issues a warning and ignores the remainder of the string.)

Taking The 1000 Genomes Project as an example, ([14], Fig. 4) shows some sequence lengths are much more common than others. In Section Results we report tests on paired end data comprised of 36 bases per end and of 100 bases per end. Both are common in The 1000 Genomes Project (in fact the most popular is 101 bases).

### Alternative tools

Highnam et al. [15] report the accuracy of four popular CPU based alignment tools. Three are open source Bowtie2 [16] bwa and BWA-MEM [17] whilst Novoalign3 is commercial). They says the tools’ accuracy lies between 91% (Bowtie2) and 98% (BWA-MEM). BarraCUDA is towards the top of this range, see “GCAT” data in the last column in Table 3. Lenis and Senar [18] tuned performance for four open source CPU aligners (Bowtie2, BWA-MEM, GEM [19] and SNAP [20]) on a 64 core AMD Opteron Processor 6376, 128 gigabyte computer. They report ([18], Table 4) BWA-MEM and GEM 3.0 give similar speed but GEM 3.0 is the fastest of the four at 383 000 sequences per second. (Notice this is for single ended 100bp next generation DNA sequences, NGS, see list of abbreviations).

Luo et al. [21] compare SOAP3-dp [22] and their own MICA on what was at the time the world’s fastest computer. The Tianhe-2 contains 48 000 Intel Xeon Phi 31S1P many integrated core (MIC) co-processor boards. They report that SOAP3 [23] running on GTX 680 [24] is the fastest of the 13 open source aligners they benchmarked. (nVidia’s GTX 680 GPU has 1536 1.06 GHz cores and they claim a memory bandwidth of 192.26 GB/s. Luo et al. give performance for MICA, SOAP3-dp, SOAP, Bowtie2 (3 settings), bwa, SeqAlto [25] (2 settings) CUSHAW2 [26] and GEM (3 Settings)). Luo et al.’s Table 3 [21] suggests that SOAP3 on a GTX 680 processes 45 500 paired end simulated NGS DNA sequences per second. However the accuracy (sensitivity) of 97.77% is the lowest of the 13 tools in ([21], Table 3) whereas SOAP3-dp (99.66%) is the highest. Apart from their own MICA, the other ten tools are all benchmarked on Intel i7-3730k, 6-core 3.2 GHz CPUs. For example (depending upon parameter settings), ([21], Table 3) suggests GEM processes from 14 400 to 20 100 sequence/sec.

nvBowtie https://nvlabs.github.io/nvbio/nvbowtie_page.html was written by nVidia on top of their nvbio CUDA templates to emulate Bowtie2 on their GPUs. They claim similar accuracy and on real 100 bp paired end NGS data they claim a single dual K80 Tesla gives a 2.1 speed up compared to Bowtie2 using 20 threads on an Intel Xeon E5-2690 v2 CPU.

The above tools are based on compressing the reference genome into a prefix index using the Burrows-Wheeler transform (which gives bwa its name). This has the advantage that typically, e.g. for human data, the whole index can be fitted into a GPU’s memory or indeed into many laptops and personal computers. However it means at best finding each NGS short read (of *n* bp) takes *O*(*n*) time. (Typically, where the NGS data are noisy or the DNA truly differs from the reference genome, e.g. due to SNPs or indels, these tools back up and perform some type of heuristic guided truncated tree search of the index. This can greatly increase run time.) Arioc [27] takes a different approach.

Arioc [27] uses hash techniques. In principle hashing allows constant time (*O*(1)) access but in practise search is complicated by the need to deal with inexact matches and the size of the hash table. Wilton et al. [27] say for the human reference genome Arioc needs about 65 GB of RAM on the host computer. Sixty five gigabyte is far more than current generation GPUs and so Arioc takes care to optimise loading it in parts into GPUs. They compare Arioc performance against four Burrows-Wheeler based tools, two CPU based (Bowtie2 and BWA-MEM) and two GPU based (SOAP-dp and nVidia’s NVBIO from http://nvlabs.github.io/nvbio/) Wilton et al. [27] run the three GPU based aligners on an nVidia K20 Tesla. They use a 6 dual 2.93 GHz cores (24 threads of execution) workstation with 144 GB of system memory for Bowtie2 and BWA-MEM. They say “Arioc demonstrated up to 10 times higher throughput across a wide range of sensitivity settings.” ([27], Fig. 8) suggests Arioc can process well in excess of 200 000 100 bp paired end simulated NGS DNA sequences against the YanHuang genome per second, although speed falls by more than a factor of ten to increase accuracy from ≈ 93.4% to ≈ 95.6%. Surprisingly ([27], Fig. 8) suggests BWA-MEM accuracy is at best 93.2% which is well down on other benchmarks, e.g. those reported by Highnam et al. [15].

### Scalability

Typically next generation sequencing (NGS) alignment tools (such as bwa, both versions of BarraCUDA and those mentioned in the previous section) have a start up overhead. Once started, data are often processed at a constant rate. (I.e. run time is linear in number of NGS DNA sequences.) However speed may be dramatically affected by user selected options and the quality of the data. For example, some tools allow the user to force all potential matches to be reported. Naturally this can considerably reduce the aligner’s speed. Similarly poor data can force the aligner to do more internal back tracking, which can similarly reduce the alignment rate (particularly if the user changes parameters to compensate for the noisy data).

In many cases the aligners have strict memory requirements. Typically the computing hardware must have sufficient RAM to hold one or more large indexes. The size of the index typically depends upon the reference genome. Once this requirement is met, there is often little additional advantage if the size of RAM is increased. With CPU based aligners, Lenis and Senar [18] showed performances gains can sometimes be had by placing the data near the CPU using it, even if the data have to be duplicated. Usually GPU based tools use techniques such as non-paged/locked memory on the host to maximise data rates across the PCI bus. (PCI buses are often used to connect GPUs and host computers.) However PCI is typically an order of magnitude slower than CPU–RAM speeds. Therefore, except for Arioc^1^ (previous section), GPU based aligners usually copy large data structures onto the GPU only once when they start. For BarraCUDA and similar aligners this means for human data the GPU must have at least 4GB of on board RAM.

GPU based aligners (such as BarraCUDA) typically get their speed by using a GPU thread per sequence. This works well where DNA strings map directly and uniquely to the reference genome. However, e.g. when data are noisy, there may be a need for the search to backtrack. Since when backtracking is invoked is different for each query, this means each GPU thread behaves differently, which means in turn that they diverge. Modern GPUs support thread divergence transparently, however divergent threads impact the GPU’s speed. In the case of the GI version of BarraCUDA we deal with this by inserting a non-divergent high speed pre-pass. This handles most cases. DNA strings which do not map simply, fall back to the slower divergent code. Thus, as with other tools, mentioned above, in practise speed will depend upon how noisy the DNA strings are. Although thread divergence is particularly a problems with GPU based aligners, the problem of noisy data needing more complex handling affects all NGS aligners and their speed can always be adversely affected by poor quality DNA sequence data.

### Genetic improvement (GI)

Genetic improvement is the process of applying search based optimisation techniques, such as genetic programming [5], directly to software [12]. Specifically, we applied grow and graft genetic programming [28] to BarraCUDA’s GPU source code. (Full details are given in [3].)

Darwinian evolution is applied inside the computer (see evolutionary cycle in Fig. 1). A grammar describing the CUDA source code and CUDA parameters, and all legal mutations of source code is automatically generated from the manually produced program, and a population of 1000 individuals each defining a set of CUDA parameters and code mutations is created. Each is applied via the grammar to give a new CUDA kernel, which is compiled. The grammar ensures the mutant code is syntactically correct and has a high chance of compiling. Each new kernel is run on ≈ 160 000 NGS DNA strings and its answers and how long it took are compared with the original code. Kernels which produce equivalent answers and are faster than the original are eligible to be parents of the next generation of mutant kernels. The fastest half of the population are given two children each, one is created by crossover with another fit parent and one by mutation. We cycle through 50 generations. The best of the last generation is tested on millions of DNA sequences not used by the GP.

In [3] we evolved new versions of BarraCUDA’s GPU code specifically for two different GPUs (K20 and K40), however it is easier to support a single version. Therefore the two evolved versions were manually reconciled into one, which was released in March 2015. This version has subsequently been maintained in the conventional manual way. Nonetheless this does not preclude re-applying genetic improvement in future. E.g. to re-tune BarraCUDA to new hardware or new types of data, such as longer DNA sequences.

Designers of Bioinformatics and other software are frequently faced with heuristic design choices. Sometimes these can be parametrised so that the choice can be delegated to the users. This can lead to a huge number of frequently opaque command line options. In which case the software designer has to provide sensible defaults. In many cases the designer makes their best guess based on anticipated use and expectations of the computer hardware that will be available. Although best placed to make these choices at the time, pressure of other tasks can make it impractical for them to re-visit these choices should circumstances change. Where parameters were exposed, search can be used to optimise them [29, 30] and if not, new GI techniques, such as deep parameter optimisation [31], might be used to automatically revisit design choices, e.g. in the light of a new use case. In [32] we demonstrated re-tuning legacy code for six different nVidia GPUs covering several generations of their architecture. As well as the comprehensive survey in [11, 12] describes several recent demonstrations in which GI was applied to Bioinformatics tools and other software.

## Method

### Programs, DNA sequences and parallel operation under multi-core Unix

### bwa 0.7.12

bwa [17] (0.7.12-r1039 (https://github.com/lh3/bwa/archive/0.7.12.tar.gz)) was down loaded from GitHub and compiled with default settings (i.e. including support for multi-threading).

### BarraCUDA 0.6.2

For comparison, the previous version of BarraCUDA, i.e. 0.6.2, was compiled with default settings (i.e. again including support for multi-threading).

### BarraCUDA 0.7.107

BarraCUDA (0.7.107), was down loaded from SourceForge (http://sourceforge.net/projects/seqbarracuda/files/latest/download). Again it was built with default setting (including support for multi-threading). However a second version was built specifically for the GT 730 which was compiled with -arch 2.1 to support compute level 2.1 (the default is now 3.5 or higher).

### Reference Genome: UCSC HG19 ucsc.hg19.fasta.gz

The reference human genome [33] was downloaded from the Broad Institute’s GATK resource bundle (version 2.8/hg19). It was converted into two indexes. BarraCUDA 0.6.2 converted ucsc.hg19.fasta.gz into an index for itself. Secondly BarraCUDA 0.7.0 converted it into an index for itself and for bwa.

### base pairs: 1000 Genomes project

One of The 1000 Genomes Project [34]’s normal (i.e. not color space encoded) paired end data with 36 DNA bases per end was chosen at random (ERR001270^2^). It contains 14 102 867 sequences. Approximately 5.7% of sequences occur more than once.

### base pairs: GCAT Benchmark

We used BioPlanet.com’s GCAT [15] 100 bp-pe-small-indel alignment benchmark (gcat_set_037, available via http://www.cs.ucl.ac.uk/staff/W.Langdon/ftp/gp-code/www.bioplanet.com/gcat/gcat_set_037). It contains 5 972 625 paired end (100 base) sequences. (Less than 0.1% of sequences were repeated.) (We have compared other nextgen tools on GCAT for this journal [35].)

#### **Example 1**

Example bash command line using process substitution, pipes and input-output redirection to run two “aln” processes (one per paired end) in parallel with “sampe”, thus avoiding use of intermediate disk files.



**Fig. 2:**
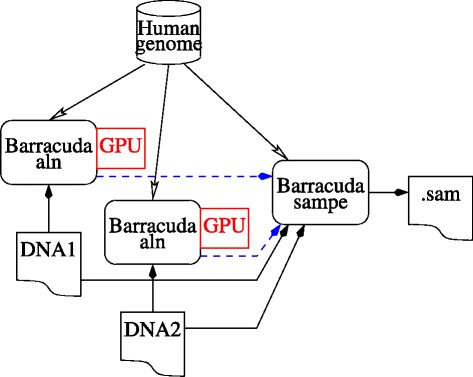
Processing paired end DNA sequences. “aln” is run two times (once per end), potentially in parallel, and its alignments are piped or passed via intermediate.sai files (*dashed blue arrows*) into “sampe” (sam (pe) paired end). “sampe” may be run in parallel. It also reads the index of the reference human genome and both ends of each DNA sequence in order to give the combined alignment in sam format. In the case of BarraCUDA, the two “aln” process each use a GPU and “sampe” uses multiple host threads. For bwa “aln” uses multiple host threads but “sampe” is single threaded

$exe1, $hg19, $seq1, $seq2 and $sam are the names of bash environment variables. $exe1 is the program, (i.e. bwa, BarraCUDA 0.6.2 or BarraCUDA 0.7.107), $hg19 is the location of the reference genome index, $seq1 and $seq2 are the files holding the pairs of DNA sequences and $sam is the output. See also Fig. 2.

## Results

bwa, the original BarraCUDA (i.e. version 0.6.2) and the GI version of BarraCUDA (i.e. 0.7.107) were each run five times on both the fourteen million real world paired end DNA sequences from The 1000 Genomes Project (“36 base pairs: 1000 Genomes project” section) and the almost six million GCAT paired end DNA sequences (Section ‘‘100 base pairs: GCAT Benchmark”). bwa was run on 12 core 2.60 GHz servers (see Table 2) whilst BarraCUDA was run on three GPUs, stretching from £50 low end GT 730 to the top of the range K80 Tesla (see Table 1). The results are summarised in Table 3.

**Table 2:** Computers. The desktop computer houses one GT 730. The servers are part of the Darwin Supercomputer of the University of Cambridge and hold multiple Tesla K20 or K80 GPUs

Type	Intel x86	Effective cores	Clock	Memory
Desktop	Core ^TM^2 CPU 6700	2	2.66 GHz	4 GB
Darwin	Xeon CPU E5-2630 v2	12	2.60 GHz	62 GB
NVK80	Xeon CPU E5-2670 v3	24	2.30 GHz	125 GB

**Table 3:** Mean number of paired end sequences processed per second

Prog	length	12 core server^a^	GT 730^b^	2 × K20	K80		Accuracy %
bwa	36 bp	1900±50	–	–	–	Mapped reads	82.05
bwa	100 bp	4500±20	–	–	–	GCAT	98.91
0.6.2	36 bp	–	3270±2 (1.7±0.05)	5300±110 (2.8±0.10)	6500±180 (3.4±0.13)	Mapped reads	83.17
0.6.2	100 bp	–	1860±4 (0.4±0.002)	8700±140 (1.9±0.03)	11700±100 (2.6±0.02)	GCAT	97.49
0.7.107	36 bp	–	7600±6 (4.0±0.11)	12900±160 (6.8±0.20)	19900±500 (10.5±0.39)	Mapped reads	83.01
0.7.107	100 bp	–	2100±14 (0.5±0.004)	8800±70 (2.0±0.02)	12800±270 (2.8±0.06)	GCAT	98.43
Improvement ratio Barracuda 0.7.107 over 0.6.2							
	36 bp	–	2.32±0.003	2.43±0.06	3.07±0.11	Mapped reads	–0.16
	100 bp	–	1.13±0.01	1.00±0.02	1.09±0.02	GCAT	1.60

In (brackets) speed relative to bwa 0.7.12. ± gives standard deviation estimated from five runs. There was almost no variation in mapping rate or accuracy reported by GCAT

^a^2.60GHz, see “Darwin” in Table 2

^b^Estimated for two GT 730 GPUs

Apart from the low end GT 730, BarraCUDA is typically between two and ten times faster than bwa on a 12 core compute server. Table 3 shows the new version of BarraCUDA is up to three times faster than BarraCUDA 0.6.2 on the real world DNA sequences (36 bp) and typically about 10% faster on the longer benchmark strings (100 bp).

bwa “aln” (via pthreads) uses multiple threads. However bwa “sampe” does not support multiple host threads. This may explain why bwa performs relatively badly on the short 36 bp 1000 Genomes Project data.

We have used large real world and benchmark sequences. However both bwa and BarraCUDA are sensitive not only to the length of the DNA sequences but also how noisy they are. Resolving ambiguous matches caused by noise slows them down.

## Conclusions

Depending upon examples, even a £50 GPU running BarraCUDA can be faster than bwa on a twelve core 2.60 GHz server. With a top end nVidia Tesla GPU, BarraCUDA can be more than ten times faster than bwa on a 12 core server.

## Endnotes

^1^ Arioc’s index considerably exceeds current GPU memories and care must be taken to try and limit the volume of re-loaded data.

^2^ ERR001270 is available from The 1000 Genome Project’s FTP site. Additionally a copy can be down loaded from http://www.cs.ucl.ac.uk/staff/W.Langdon/ftp/gp-code/barracuda_0.7.105/1000.

## Abbreviations

aln: Alignment process used by both bwa and BarraCUDA
bp: Base pair
BWA: Burrows-Wheeler algorithm
BarraCUDA: A parallel computer program which uses nVidia GPUs for aligning DNA particularly sequences produced by short next generation DNA scanners against a reference genome, particularly the human genome
bwa: A program for aligning DNA particularly sequences produced by short next generation DNA scanners against a reference genome, particularly the human genome
C: A computer programming language
C++: An object orientated computer programming language based on C
CUDA: Computer uniform device architecture. A set of software tools to enable programs (particularly non-gaming software) to run on nVidia GPUs
GATK: The Genome analysis toolkit suite of computer software written by the Broad Institute
GB: Gigabyte 1 073 741 824 bytes
GCAT: A DNA alignment benchmark
GHz: Gigahertz, 10^9^ clock ticks per second
GI: genetic improvement of software
GPU: Graphics processing unit
GPGPU: General purpose computing on GPUs
GT 730: A domestic gaming GPU designed by nVidia
indel: A DNA mutation which inserts or deletes DNA bases, so changing the length of the DNA sequence
K20, K80: Types of computational accelerators designed by nVidia based on their parallel graphics cards (GPUs)
nextgen: Next generation (see NGS)
NGS: Next generation DNA sequence
SAM: Sequence alignment/map computer file format
sampe: A software process which resolves pairs of paired end DNA alignments produced by earlier “aln” processes and converts them into SAM text file format
